# The Advantages of Nitrous Oxid Gas and the Proper Manner of Administering It

**Published:** 1905-09-15

**Authors:** S. Straith

**Affiliations:** Detroit


### THE ADVANTAGES OF NITROUS OXID GAS AND THE PROPER MANNER OF ADMINISTERING IT.

BY S. STRAITH, D.D.S., DETROIT.

Read before the Michigan Dental Association, July 10, 1905.

In attempting to write upon the subject of nitrous oxide gas, confining myself particularly to thoughts touching upon the specific advantages of gas as an anesthetic and the best or proper manner of administering it, necessarily brings gas, theoretically at least, in comparison and competition with all the other anesthetics, chloroform and ether, as well as some other general anesthetics, and all the *local* anesthetics. The element of safety, or rather its converse, *danger*, should contra-indicate the use of chloroform or ether for any surgical work the dentist is called upon to perform unless efforts to use a safer anesthetic have proved ineffective. Then this statement leads us at once to the consideration of the one *pre-eminently* important point in the choice of *any* anesthetic, namely its safety.

We too often forget that in the administration of an anesthetic a human life is at stake. Notice the anxiety of a parent when his child is placed upon the operating table or in the operating chair. Has it ever been your lot to test your nerve for such an ordeal? If not, then it is impossible for you to appreciate the feelings that such an occasion prompts. Notice that all the questions or petitions asked by the patient or his friends are touching upon the safety of the anesthetic used and not the convenience, cost or personal likes or dislikes, by which, I fear, too often the dentist justifies himself in the belief that the anesthetic of his choice is the one indicated. If, then, we are to consider *safety* as one of the advantages of nitrous oxide gas, to what do we or shall we turn for evidences of its safety. We know that gas was the first anesthetic discovered, and we also know that, so far as statistics help us, we are told that less than ten deaths have occurred from its use, or from the operation when gas was the anesthetic used. Then when you deduct from this number the two or three upon whom a post-mortem revealed a portion of a mouth prop or a part of a denture lodged in the respiratory tract causing asphyxiation, and then the others to whom chloroform or ether had been given *with* the gas, you have nearly robbed gas of its stigma of being a death dealing agent. We all know that statistics are dry reading and quite as dry to command a listening and attentive ear, yet they are the necessary proof in many an emergency. I have in my possession the record of about 500,000 administrations of gas in three different offices without any serious results of any nature. To use this record in comparison with the record of any other general anesthetic, leads to only one conclusion, viz, in the language of Dr. Squibb: "Gas is the most important of all anesthetics, because destined to relieve a greater aggregate amount of pain, with greater safety than any other agent.'' And I think he might have said, than all other agents *combined*.

A comparison of the safety of gas with other anesthetics viewed from merely the number of administrations, is very unfair to gas. When you consider that the other general anesthetics best known and generally used, are used by men who have had good training and instruction in their use, and that gas is given by men indiscriminately, the large majority of whom never administered it under a preceptor, and that it is given hundreds of times by these same inexperienced men to patients who were forbidden *any* anesthetic by men well qualified to judge of their physical condition, then any such comparison is unfair to gas. The successful results from the use of gas in the hands of so many untrained and incompetent persons is one of its strongest points as an advantage in its use, because it is an almost absolute guarantee of its safety.

In considering the advantages to be gained or realized from the use of gas, I find that many things *I* count as "gain" others count "loss." When asked his objection to gas, one says "I do not like the color of the patient." Another says, "The patient's friends get nervous." Another, "There is too much noise." Another, The anesthesia is too short," etc., etc. To the first objection I answer that cyanosis is not always present in the administration of gas, and when it is observed it is nothing that should frighten the operator, for we know that the moment the gas is shut off and air is admitted cyanosis gradually disappears. Rather than an objection, I sometimes count the cyanosis an aid to me in the administration of gas, for it tells me I am approaching the danger limit. However, the appearance of cyanosis is never the signal to cease the administration of the gas unless the reflexes are gone, for I never begin an operation until that condition is present. I cannot quite understand why cyanosis is considered such a contra-indication to the use of gas; for when caused by the inhalation of gas, we know that the admission of air will restore the normal color. It has never failed yet and never will. If you were so unfortunate as to sever the radial artery you would not hesitate to bandage your arm shutting off the supply of arterial blood because your hand would become cyanosed.

To the second objection, I answer that the operating room is no place for the friends of the patient during the operation but when they are present, then their position should be *behind* and *away* from the operating chair, so that any unpleasant or unusual symptoms or appearance of the patient will not be noticed by them.

As for the noise, I know that I do not have the amount of screaming that I did when I used a local anesthetic, besides I have the satisfaction of knowing that the patient either does not realize he is screaming or does not know why he screams.

The improper administration of gas has done more to bring it into disfavor with the profession, as well as the laity, than the inefficiency of the operator. First of all we should use *pure* gas. This, I fear we do not always have in the cylinder gas. Then our appliances for administering the gas should be perfect and in perfect working order. I have often inspected the appliances of dentists who were not as successful as they wished to be in the use of gas and came to the conclusion that very few could have done better services than they with the appliances at hand. The face piece should so fit the face as to completely exclude all air. The tubing should be plain *uncovered* rubber. The covered tubing in common use has such a thin rubber tube that it very soon breaks and then admits air. See that your mouth prop, if small enough to be swallowed, is made secure to a string, so it may be easily withdrawn from the mouth should there be any attempt at swallowing it. Remove all dentures. It is my practice to exclude all the air possible, while administering gas, unless from extreme nervousness of the patient or an apparent difficulty in breathing, when a breath of air will relieve the patient and restore his confidence. I know this is contrary to the practice of some others who advocate admixture of air with the gas, but my experience with diluted gas has not been satisfactory to me or the patient. I believe the most common and worst mistake of gas anesthesia is *incomplete anesthesia*. Too many dentists, for one reason or another, cease the administration of gas at the first signs of anesthesia—just when the anesthesia should be pushed. The result is an incomplete anesthesia and the patient, although, as a rule, unable to offer resistance, realizes what is taking place and regains consciousness before the operation is completed. This is very unsatisfactory to the patient and does not increase his admiration for the dentist or his method of relieving pain.

Scarcely a day passes that I do not hear a patient say, "Doctor, please give me enough gas, for the last time I took it, I felt the pain and knew just when he extracted the tooth." There are only two things that can cause such a result, if there is no trouble in extracting the tooth, and they are—insufficient gas or too much air with the gas.

Then, do not be in a hurry to awaken your patient. Nearly every patient has a dream while under gas anesthesia. This dream may be of a very pleasant or a violent character, and if it should be the latter you may have some trouble to control your patient should you hurry the awakening.

The time has not come when we have an anesthetic to which no objection can be made in any instance, and, although to some it may be the wrong position to take, I am satisfied to leave the experimenting to the other fellow, and use the anesthetic that I believe insures me the greatest amount of safety to my patient.

### DISCUSSION.

DR. G. H. BROWN: It is always natural for us, when some one else speaks, as we should speak to think that he is right, therefore, I most cordially endorse what the essayist has said. His paper covers the ground almost exactly as I have experienced it after twenty-five years' use of nitrous oxide. It is a great wonder with me to-day that the profession does not use nitrous oxide gas more liberally. After over twenty-five years' experience I have this to testify, that it is my honest belief that no deliterious effect has ever come from the intelligent use of nitrous oxide gas. When fatal cases have occurred it has been either through poisonous gas, by an over-heating and making a nitric oxide gas instead of a nitrous oxide gas, or by the carelessness or ignorance of the operator. I honestly believe to-day that in the hands of an intelligent average man, nitrous oxide gas is abolutely safe, and can be administered to almost any patient. While I absolutely refuse to give it to some patients, whom the eye at once tells me are in an unhealthy condition from one cause or another, those patients should not be given any anesthetic unless for major operations where absolutely necessary. To illustrate, I will relate one experience I had with a patient, a servant girl, a large, healthy Scotch girl, who came to my office one day to have all her front teeth extracted. She said she would like to take gas but did not know much about it, but would like to try it and have one or two teeth extracted. I examined her heart, and so far as I could determine, she was in a perfectly healthy condition. I put her in the chair, gave her the gas and extracted two or three teeth just to satisfy her. She got up without any trouble at all, and was perfectly delighted with the operation. I am telling this to illustrate the first statement that I made when I said that all fatal results from nitrous oxide gas were due to either carelessness or ignorance of the operator, or to impure gas. This same patient came back to me the next day. I had had such a pleasant experience the day before with her, that I paid no particular attention to the patient, but placed her in the chair and with the assistance of my assistant, administered the gas. There were several teeth and roots to be extracted and as I felt perfectly safe, I made no examination of the heart, I carried her fully under the influence of the gas and I picked up my instruments and went at the operation and extracted all the teeth. I regret to say that I paid very little attention to the patient, for I was very busy with the operation. The moment I had completed the operation I turned to the patient and she was black. What was the matter? The patient at that moment was practically dead. I had neglected the patient and her tongue had fallen back into her throat and she was smothering. If left for one minute more she never would have recovered. If I had become excited and gone for a physician, she never would have recovered. I instantly thrust my finger in and brought her tongue forward, gave the chair a kick and let her head forward to the floor, I set my assistant to work at artificial respiration and dashed a glass of water into her face, and in a half minute she took a long breath and recovered. Before she recovered her consciousness or knew anything about it, she was in a normal condition. She never knew, and does not now know that she was in any danger. This simply illustrates to you that we can do harm bycarelessness. If that patient had died on my hands I should have been entirely to blame for it. Probably the law would have excused me, but my own conscience would have told me that it was my own carelessness in carrying that patient too far with the anesthesia. This is, I believe, the history of nitrous oxide gas administration. I believe in ninety-nine cases out of a hundred it is absolutely safe, and if properly and intelligently handled, there should be no danger.

Dr. N. S. Hoff: I do not think there is any question in the mind of any one who has had anything to do with the administration of anesthetics as to the place in regard to safety which nitrous oxide occupies as a general anesthetic. As Dr. Straith has well put it, it is as nearly an absolutely safe anesthetic as we have. I do not think it will be necessary for me to waste any more time to try to prove that, but I could add testimony to what has already been said as proof. The fact which discredits it is that it is such a difficult agent to manipulate properly. The anesthesia produced by it is for such a brief period that many operations cannot be well made with it—extractions, such as impacted teeth or teeth which are difficult to extract, and other surgical operations about the mouth cannot be successfully made with it. It is difficult in some cases to control the patient. In many operations that we desire to make, we want to have the patient quiet. Very often the temperament of the patients is such that they are violently excited under nitrous oxide, this is too often brought about, however, by insufficient administration of the gas. Men, who have not studied and informed themselves carefully about the physiological action of nitrous oxide gas often get scared by things which a man, who understands the conditions and the influence the anesthetic has upon the patient, would not take into account at all, but would pass the patient on into that period of quietness where the operation can be made successfully. These common experiences have discredited nitrous oxygen with the dental profession, the briefness of the anesthesia and the difficulty of controlling the patient when it is absolutely necessary to have the patient quiet so that the operation can be made satisfactorily. It is an objection to nitrous oxygen that it requires considerable apparatus, that cannot be kept in order so that it is always ready to be used. There is no gas apparatus on the market that will take care of itself, and every time the apparatus is used it should be inspected so that it shall produce the proper result. The most serious objection to nitrous oxide is that it is so brief in its physiological action, and the effect is produced so suddenly that the patient is brought so nearly to that condition where it is only a step into the dangerous condition, that it frightens us when we use it; hence we do not carry the gas far enough to produce the satisfactory anesthesia. Dr. Straith states in his paper that he does not mind the cyanosis. I think we should note it carefully; it is one of the symptoms in the use of ether and chloroform that the administrator always takes into account. While I am not satisfied from my own study and experience with it that the cyanosis that we get from nitrous oxide is the same as that produced by ether and chloroform, it is sufficiently alarming as a symptom that we can't afford to ignore it. We should not produce the excessive cyanosis or blackening in the face which has been referred to above. An experienced operator once said to me he never attempted to perform an operation until the patient was black in the face. A condition of that kind means a complete change in the blood which will not support or sustain life; and is a condition which would very easily interfere with or destroy the nervous functions of the brain centers. It certainly should not be maintained for any great length of time.

I do not know any other general anesthetic that can be more safely or effectively used. If in administering an occasional inhalation of air be permitted or it be diluted with oxygen, the cyanosis will be prevented and a quieter anesthesia result. It is easy to attach an oxygen cylinder to the modern gas apparatus by means of which the intense cyanosure can be prevented. I regard as of more consequence than Dr. Straith seems to indicate the cyanotic condition. I certainly should not feel easy and comfortable if I produced in my patient anything approaching that of "a *face as black as a hat.*" We can get not only complete anesthesia without cyanosis, but we can get longer anesthesia with nitrous oxide and oxygen and without danger of harming our patient. Although it may be a little more troublesome to administer gas with oxygen, I think that we will find the results so much more satisfactory in securing quietness and prolonged anesthesia without danger that it will justify the additional trouble of adding the oxygen.

DR. HAMPTON: I have nothing but good words to say of nitrous oxygen. I would like to ask Dr. Straith how long we may continue it with the nasal apparatus. Many times we wish to continue the anesthetic and our work demands longer time, and I wish to know how long we can safely keep the patient under the nitrous oxide.

DR. STRAITH: There seems to be a hesitancy on the part of a good many to give gas to everyone; and Dr. Brown says he makes an examination of some people before giving it to them, and absolutely refuses to give it so some. Now, it is far from my intention to have any one believe that I wish to be careless in the administration of gas. I also want you to just as distinctly understand that I am absolutely in favor of gas, and have so little fear of it that any person who has life enough to come into my office can take gas at my hands. I have given it to hundreds of patients who have come to me and said that their physician had forbidden them to take any anesthetic. I know one case in particular: A lady had twelve or thirteen teeth to be extracted. Her friend came to me first and said: "This lady has heart trouble so badly that she drops over a half dozen times a day at her work. She is willing to take the anesthetic, but she wants you to know her condition before she takes it." She painted the case to me so black that I refused to give the anesthetic unless she would have the physician come in and examine her. I would not take it upon myself to pass upon the physical condition of the patient. The doctor came and he said, "I think this is more nervousness than anything else." I gave the gas and removed the teeth and never had a patient act better in all my experience. That moment I said: "I shall never refuse to give gas to any patient that comes into my office." I have given it thousands of times and I have never refused to give it to a person yet and do not expect to refuse—just because I have so great confidence in gas; it has been with me so successful. I have not done everything with it that I wish I could do, but I have done a good deal with it and I have done some things with it that Dr. Hoff says are difficult to do. I have with me to-day the models of a few lower molars that I took out in which I was successful generally with the first administration. One man came to me on his sixty-ninth birthday to have the roots of a lower second molar removed, and, going after them, I uncovered the third molar. I gave the man gas three times and got the third molar out at the third administration, in less than thirty minutes. Now, if this cyanosis is caused by robbing the blood of the oxygen and excluding the air, then our blood is not oxidized and the blood in circulation is all venous; we know that if we admit the atmospheric air and the lung gets some of it, the blood will clear up. While some say anesthesia is due wholly to asphyxiation, I do not agree with them. We do not understand just how nitrous oxide anesthesia is produced, yet we know it is produced and we are of one accord that the cyanosis is produced by the de-oxidation of the blood. If this is true, then the admission of atmospheric air will clear it up. If that be the reason we have cynosis, why is it so dangerous? A short time ago I gave gas to a lady upwards of seventy years of age, who was in a physically bad condition, and a surgeon removed her eye, which took eight minutes. The Surgeon told me that he would not dare to give chloroform to the patient; he had tried a local anesthetic the week before and it did not work. He told me he never had a more successful anesthesia. The patient within five minutes from the time he was through was able to go back to her room and was talking to a friend. I have had a number of cases since I came to Detroit, which have lasted twenty minutes, and there was scarcely any cyanosis.

I have noticed the records of the length of anesthesia produced by the admixture of air and oxygen and have failed to see the record of a longer anesthesia than I think I have had with pure gas. In regard to the bulkiness of the apparatus which we must use and the difficulty in keeping it in repair, I would say that I am called quite often to go to homes and hospitals to administer gas, and I have a small apparatus that contains a cylinder of gas and a bag which I can take with me in my valise with a face piece, and can give it from the little gas bag without trouble. I do not think when the safety of our patient is at stake that we should consider any little inconvenience to ourselves which will insure absolute safety for our patients.

The experience that Dr. Brown had with the tongue falling back into the throat I have had once or twice. I try to keep my patient as nearly as possible in an upright position in the chair. I do not want them leaning back in the chair and the tongue is not then so likely to go back in the throat, nor the teeth fall down the throat.

In answer to the question as to how long we dare proceed with anesthesia with the nose inhaler, I confess to you I do not know about the nose inhaler. I bought the Hurd apparatus just before I came to Detroit and gave this apparatus a good trial; in my hands it is not as successful as an inhaler that covers the nose and mouth, there are cases, of course, where we cannot use it. I prefer to use a face piece, that goes over the nose and the mouth. In that case we cannot continue the anesthesia any longer than the initial anesthesia. Where I can have the face at my disposal, I do not care how long you want anesthesia, I can give it to you with gas. I take the inhaler off when I get labored breathing and give the patient a breath of air. Sometimes there is a little headache following the operation, but not as much as under other anesthetics. In some cases the patient is ready to get up and walk away as soon as the surgeon is through, and experiences no difficulty.

Gas does not act well where liquor and stimulants have been taken. Some people have a mistaken idea that they must brace up to have a tooth out. When they come to me with too much of that, I tell them to go away and sober up before they can have gas at my hands. I have had one or two experiences where people have given me all the "time" I wanted while they were in the chair, and that is one reason why I mentioned in my paper not to be in a hurry to awaken your patient. If the patient has been drinking it will bring out either his good qualities or his bad ones. Another condition that if I knew existed I should be a little wary of, that is the tobacco habit. I had a patient, the son of a physician, who was an inveterate user of cigarettes. I did not know it until after I had removed a lower third molar. In the administration of the gas he was quite hard to hold in the chair, and had the father not been present and given me his help I could not have managed the boy. As soon as he got violent I took the gas away, and when he quieted down I gave it to him again. I gave it three times before he finally succumbed. I said to the young man afterwards: "You are either a drinker or a user of tobacco." His father told me afterwards that he had been almost an inveterate cigarette fiend. Tobacco and liquor contra-indicate the use of gas.

